# Emergency Pneumonectomy: A Life-Saving Measure for Severe Recurrent Hemoptysis in Tuberculosis Cavitary Lesion

**DOI:** 10.1155/2015/897896

**Published:** 2015-01-05

**Authors:** Ravisagar Patel, Abhinav Singh, Rajendra Mohan Mathur, Anula Sisodiya

**Affiliations:** Department of Cardiothoracic and Vascular Surgery, SMS Hospital, 177, Ram Gali No. 2, Raja Park, Jaipur, Rajasthan 302004, India

## Abstract

Hemoptysis constitutes a common and urgent medical problem. Swift and effective management is of crucial importance, especially in severe, life-threatening cases. Because of bronchial artery or a branch of pulmonary artery erosion due to cavitary infiltration, bronchiectasis, fungus ball, broncholithiasis, or destroyed lung, the bleeding can lead to highly compromised gas exchange or sometimes can be a life-threatening situation. Chest computerized tomography and bronchoscopy remain the methods of choice for lateralization of the disease. Some patients can be treated successfully with endobronchial interventions. Bronchial artery embolization can be rewarding in some patients but the recurrence rate is higher in tuberculosis than other etiologies of hemoptysis. Surgical resection of the lung, mainly lobectomy, remains a life-saving procedure but it should be performed very selectively to avoid higher postoperative morbidity and mortality.

## 1. Introduction

Hemoptysis is the expectoration of blood originating from the lower respiratory tract. Most cases are minor and treatable or self-limiting. Hemoptysis in a patient with tuberculosis is not an unusual condition, especially before the antibiotics are administered. This complaint reduces by time with treatment. However, the amount of bleeding can be very high and some patients are lost due to this massive or major hemoptysis. The definition of severe or massive hemoptysis varies but is usually defined as the expectoration of 300–600 mL of blood in 24 hours or bronchial blood loss that causes hemodynamic or respiratory compromises [1–3]. Massive hemoptysis constitutes 1–1.5% of all hemoptysis cases and can be life threatening either as a result of compromised gas exchange or because of circulatory collapse secondary to acute blood loss [3]. Without appropriate treatment, life-threatening hemoptysis has a mortality rate of up to 50–100% [4, 5]. Clinical examination and chest X rays can be helpful in determining the bleeding side but bronchoscopy combined with imaging technology usually identifies the bleeding site in the lungs more precisely. Still in 15–20% of cases the cause of hemoptysis cannot be fully determined [2, 6]. When diagnostic tools fail to identify the source of bleeding, severe hemoptysis becomes an emergency because failure to contain it can lead to death. Bronchial arteriography and bronchial artery embolization (BAE) may provide an effective means of rapid diagnosis and treatment of such medical emergencies [1, 4, 6]. However, the long-term success rate of BAE is known to be unfavorable. Long-term recurrence rates are reported to be from 10 to 52% [7]. Gourin and Garzon have recommended prompt surgical resection for patients having more than 600 mL blood in 24 hours [8]. For such a patient mortality rate is 18% by surgery as compared to 75% rate in those treated conservatively.

### 1.1. Etiologies of Hemoptysis in a Tuberculosis Patient

Bronchial artery is the major cause for bleeding in most patients with major hemoptysis. However, in a patient with tuberculosis, the erosion of a “Rasmussen aneurysm” (dilatation of pulmonary artery branches due to chronic inflammation in a tuberculosis cavity) may be responsible for hemoptysis. Chronic inflammation of bronchial walls in tuberculosis bronchitis may cause destruction and, as in the case of bronchiectasis, may lead to bronchial artery bleeding. Since bronchial arteries have higher pressure than the pulmonary arteries, such bleeding may also be severe and difficult to control. When a tuberculosis cavity invades parietal pleura and chest wall, erosion of intercostal arteries or subclavian or internal mammary arteries may also be associated with hemoptysis. Development of fungal infection in old tuberculosis cavity is another important cause of hemoptysis. Aspergillum species are most commonly the causative organism. Intracavitary mycetomas may be seen with either of these infections. Broncholithiasis is development of calcium deposits on peribronchial lymph nodes during healing process of chronic granulomatous condition, most commonly tuberculosis. Erosion of bronchial wall and peribronchial arteries by broncholiths may be another cause of severe hemoptysis.

## 2. Case Report

**Figure 1:**
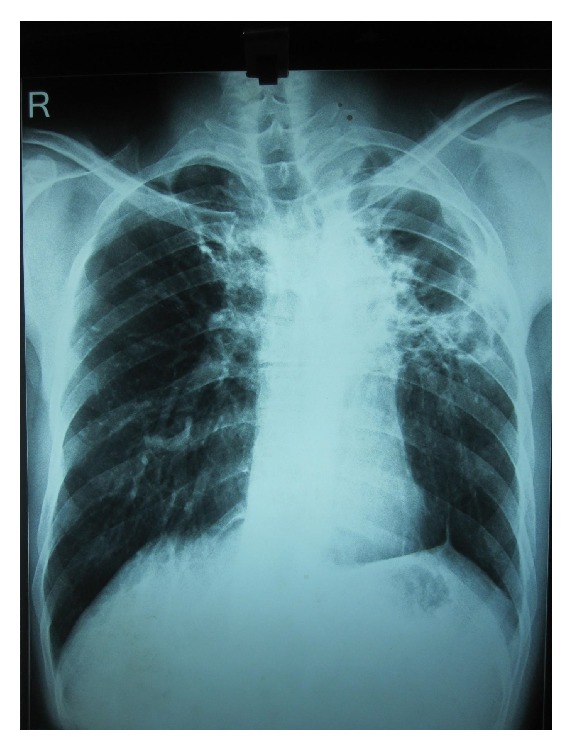
Chest X-ray showing multiple cavitary lesions of left upper lobe, with fibrosis of mediastinum causing deviation of trachea to left side and tenting of left lobe of diaphragm.

**Figure 2:**
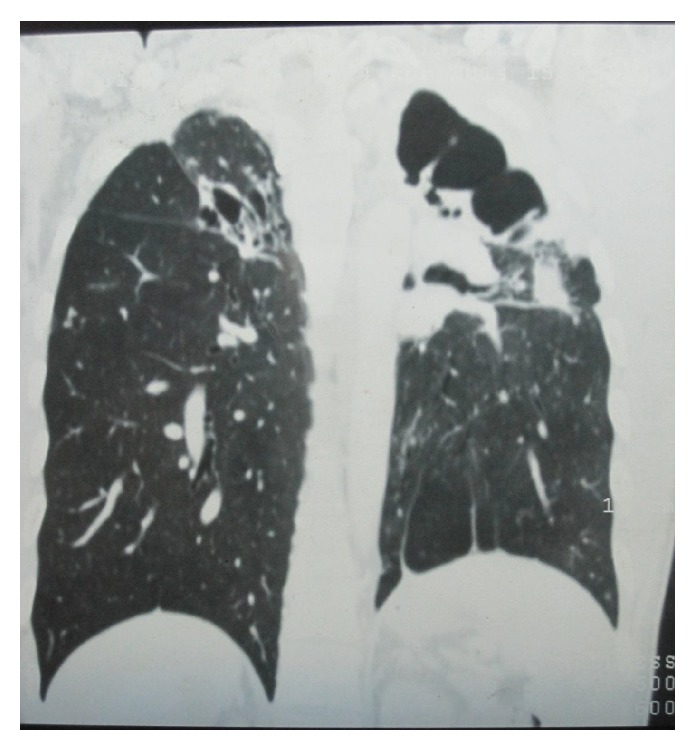
CT scan thorax showing multiple cavitary lesions in left lung predominantly in upper lobe, with hemorrhagic debris in the cavities. Bronchiectatic changes were also noted in right lung but to a milder level of affection.

**Figure 3:**
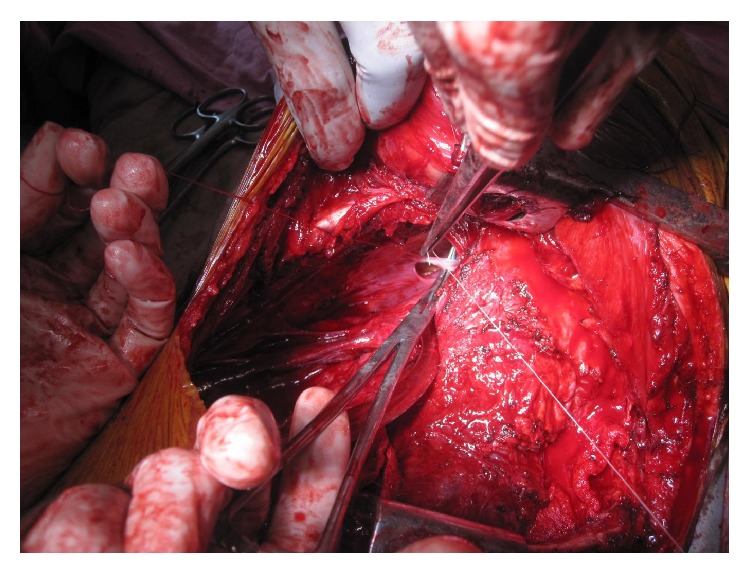
Ligation of bronchial artery being done.

**Figure 4:**
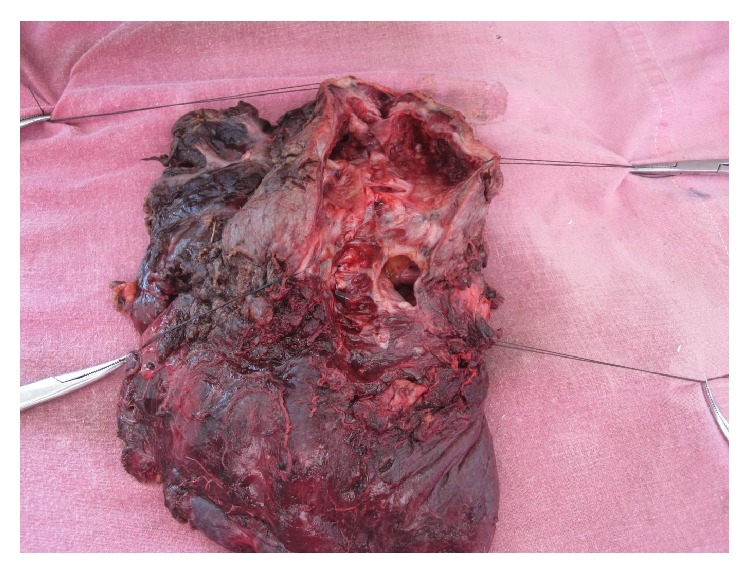
Pneumonectomy specimen cut opened to show multiple cavities in left lung in upper lobe that extended up to the lingular lobe and also some area of destroyed lung was found in left lower lobe.

This 36-year-old Indian male presented to Sawai Man Singh Hospital Emergency Room with the chief complaint of hemoptysis, having coughed up approximately 500 mL of bright red blood in the previous 12 hours. The patient was an active smoker, with a smoking habit of 45 pack-year. The patient had history of pulmonary tuberculosis 5 years back for which he had taken complete treatment. During these 5 years, the patient had few episodes of hemoptysis amongst which 2 were major episodes, for which he was admitted to the hospital and bronchial artery embolization was done for 2 times, the first one 2 years back and the second one 6 months back from this admission. On presentation, the patient had hypoxemia, tachycardia, and low blood pressure. The patient had pallor and anemia. He was admitted to the intensive care unit for close monitoring and treatment. The patient received blood transfusions because of a rapid fall in hemoglobin levels (the patient had a hemoglobin level of 12.5% on admission, which had dropped to 9.6% in a single day) and severe hemodynamic instability. Chest X-ray showed multiple cavitary lesions of left upper lobe, with fibrosis of mediastinum causing deviation of trachea to left side and tenting of left lobe of diaphragm (Figure 1). The CT scan of patient showed multiple cavitary lesions in left lung predominantly in upper lobe, with hemorrhagic debris in the cavities. Bronchiectatic changes were also noted in right lung but to a milder level of affection (Figure 2). He underwent emergency bronchoscopy to identify the site of bleeding. The bronchoscopy revealed that he bled massively from the left tracheobronchial tree. Since a definite lateralization of the bleeding source had been established, a right sided double lumen endotracheal tube was inserted to protect the right lung. The patient was nursed in the intensive care unit. Bronchial lavage cytology was negative for malignancy. The very next day the patient again bled profusely from the left lung, and at this point surgical management was deemed necessary because the exact bleeding side had been identified and the patient was in severe hemodynamic compromise. The patient was taken for emergency surgical resection of the upper lobe of left lung, and one large bronchial artery was identified and ligated (Figure 3), but, due to dense difficult adhesions all over the parietal surface of left lung, decision of pneumonectomy was made as the time to lyse the adhesions and the generalized oozing that would occur would have compromised the patient more. The stump of the bronchus was not reinforced in this case as there were strong adhesions and surrounding tissue was edematous, and there was urgency to complete the surgery due to hemodynamic instability. After resection, the left lung was cut open for inspection that revealed multiple cavities in left lung in upper lobe that extended up to the lingular lobe and also some area of destroyed lung was found in left lower lobe (Figure 4). Histopathological examination confirmed the bronchiectatic changes and culture for tuberculosis bacteria was positive.

## 3. Discussion

Diagnosis of the etiology and anatomic bleeding localization of hemoptysis is mandatory because a wide range of causes from bronchitis to malignancy can lead to hemoptysis. Massive or recurrent hemoptysis may be life threatening. Pulmonary TB is one of the most well-known etiologies for hemoptysis; however, there are many types of tuberculosis lesions which lead to hemoptysis. Tuberculosis cavities are the common type of pulmonary lesions which are liable to major and massive hemoptysis. The first report for controlling life-threatening hemoptysis by bronchial artery embolization was done in 1973 by Remy and colleagues [9]. By selective bronchial artery angiography, the site of bleeding of a bronchial artery is determined first and then application of embolization material obliterates the site of leak from bronchial artery. White and colleagues suggest that bronchial artery embolization is a palliative procedure and the potential for recurrent hemoptysis exists as long as the disease process is not cured by drug therapy or removed surgically. Recurrence after bronchial artery embolization may be related to incomplete embolization, recanalization of embolized vessel, revascularization of collateral vessels associated with the progression of the underlying disease, or bleeding coming from a pulmonary artery branch as in the case of cavitary tuberculosis.

Management of massive hemoptysis and timing of surgical intervention pose difficult problems. Emergency surgery should be reserved only for those patients (I) having adequate lung function; (II) having exact site of bleeding definitely defined; (III) continuing bleeding despite the adequate measures taken [10]. Emergency surgery performed for massive hemoptysis has always higher risk than a planned surgery. Most important reasons for increased mortality in emergency situation are the continuing bleeding during the operation and proceeding aspiration to uninvolved lung and hypovolemia. So deciding to perform an emergency surgical resection in a patient with massive hemoptysis remains a real challenge.

Resection of the lung parenchyma may lead to respiratory insufficiency. For this reason, the amount of lung resection should be as small as possible while resecting the main source of bleeding. In most cases, a lobectomy is the standard operation. Because in most cases it is not possible to define the bleeding segment, a segmental resection is rare. Many cases have microscopic disease that could not be identified on CT scan or gross examination at operating table, so a nonanatomic resection using staplers has more chances of late recurrence of the hemoptysis. In some cases, pneumonectomy is inevitable due to whole lung involvement (destroyed lung) or when the bleeding site is lateralized but not localized. In this case we had taken the control of pulmonary artery at the beginning of the surgery, as is our institution protocol for lung resections. This helps to control any torrential bleeding during surgery and if conversion to pneumonectomy is required in any case, the procedure is more controlled and safer. The complication rate is reported to increase by emergency pneumonectomy compared to emergency lobectomy (72% versus 52%)

## 4. Conclusion

Locating the site of the hemoptysis and defining the etiology may be difficult especially in case of a massive hemoptysis. Bronchial artery embolization may be a good method for controlling hemoptysis and gaining time for a planned surgery in general population. However, in tuberculosis patients the success rate of this method seems to be decreased probably due to the presence of bleeding also from a pulmonary artery branch in this group of patients. In case of massive bleeding, emergency surgery becomes inevitable but carries higher risk than a planned surgery. Surgical resection is still the definitive treatment with acceptable rate of morbidity and mortality.
